# Discovery of Novel Tryptanthrin Derivatives with Benzenesulfonamide Substituents as Multi-Target-Directed Ligands for the Treatment of Alzheimer’s Disease

**DOI:** 10.3390/ph16101468

**Published:** 2023-10-16

**Authors:** Guoxing Wang, Jiyu Du, Jie Ma, Peipei Liu, Siqi Xing, Jucheng Xia, Shuanghong Dong, Zeng Li

**Affiliations:** 1 Inflammation and Immune Mediated Diseases Laboratory of Anhui Province, Anhui Institute of Innovative Drugs, School of Pharmacy, Anhui Medical University, Hefei 230032, China; wangguoxing12@yeah.net (G.W.); dujerry123@163.com (J.D.); liupeipeiah@yeah.net (P.L.); wanglihuasan@163.com (S.X.); 13093635611@163.com (J.X.); dsh1422620270@163.com (S.D.); 2 Anhui BioX-Vision Biological Technology Co., Ltd., Hefei 230032, China; 3 Neurosurgery, The Central Hospital of Wuhan, Tongji Medical College, Huazhong University of Science and Technology, Wuhan 430074, China; sysuahmu26@sina.com

**Keywords:** Alzheimer’s disease, multi-target-directed ligands, tryptanthrin derivatives, cholinesterase inhibitory activity, neuroinflammation

## Abstract

Based on the multi-target-directed ligands (MTDLs) approach, two series of tryptanthrin derivatives with benzenesulfonamide substituents were evaluated as multifunctional agents for the treatment of Alzheimer’s disease (AD). In vitro biological assays indicated most of the derivatives had good cholinesterase inhibitory activity and neuroprotective properties. Among them, the target compound **4h** was considered as a mixed reversible dual inhibitor of acetylcholinesterase (AChE, IC_50_ = 0.13 ± 0.04 μM) and butyrylcholinesterase (BuChE, IC_50_ = 6.11 ± 0.15 μM). And it could also potentially prevent the generation of amyloid plaques by inhibiting self-induced Aβ aggregation (63.16 ± 2.33%). Molecular docking studies were used to explore the interactions of AChE, BuChE, and Aβ. Furthermore, possessing significant anti-neuroinflammatory potency (NO, IL-1β, TNF-α; IC_50_ = 0.62 ± 0.07 μM, 1.78 ± 0.21 μM, 1.31 ± 0.28 μM, respectively) reduced ROS production, and chelated biometals were also found in compound **4h**. Further studies showed that **4h** had proper blood–brain barrier (BBB) permeability and suitable in vitro metabolic stability. In in vivo study, **4h** effectively ameliorated the learning and memory impairment of the scopolamine-induced AD mice model. These findings suggested that **4h** may be a promising compound for further development as a multifunctional agent for the treatment of AD.

## 1. Introduction

Alzheimer’s disease (AD) is an age-related irreversible and progressive neurodegenerative disease which is primarily characterized by dementia, cognitive and memory loss, and language skill decline [1,2]. With the prolongation of people’s lifespans, the incidence of AD is also increasing every year, and effective treatment of AD has become a global problem affecting human well-being. Despite substantial research efforts to elucidate the pathology of neurodegenerative diseases, no effective treatments have been developed because of their complex etiology and multifactorial progression [3,4]. In addition, several hypotheses have been proposed and intensively studied involving key pathological pathways such as the cholinergic system, amyloid cascade, oxidative stress, neuroinflammation, and metal ion homeostasis [5].

The traditional cholinergic hypothesis indicates that acetylcholine (ACh) is an important neurotransmitter responsible for learning and memory, and its depletion is a key cause of cognitive dysfunction [6]. Acetylcholinesterase (AChE) and butyrylcholinesterase (BuChE) are two cholinesterases (ChEs) used for the hydrolysis of acetylcholine in the brain. The proportion of acetylcholinesterase (AChE) in a healthy adult brain is equivalent to 80% of cholinesterase (ChE), with the remainder being butyrylcholinesterase (BuChE) [7,8]. Studies have found that the importance of AChE and BuChE varies in different stages of AD, and the early and middle stages of AD primarily involve balancing the cholinergic system in the synaptic cleft by inhibiting acetylcholinesterase [9,10]. However, with the progression of AD, the level and activity of BuChE gradually increases, whereas the activity of AChE decreases [11]. Therefore, from a long-term perspective, the development of dual inhibitors of AChE and BuChE is beneficial in addressing the different pathological stages of AD [12,13,14].

In addition to the hydrolytic function of AChE, substantial evidence suggests that AChE can accelerate the aggregation and deposition of Aβ plaques and form stable AChE–Aβ complexes which can lead to cognitive dysfunction [15]. Furthermore, Aβ can serve as a donor of oxygen free radicals, generating reactive oxygen species (ROS) that directly affect the normal physiological functions of neural cells [16]. Therefore, designing ChE inhibitors that block AChE and Aβ accumulation may provide additional benefits for AD treatment [17,18]. Moreover, excess metal ions (Cu^2+^, Zn^2+^, Al^3+^, and Fe^2+^) were found in Aβ plaques of AD brains, and these abnormally high levels of redox-active metal ions catalyzed the production of ROS by participating in the formation of plaques. Excessive ROS can lead to a series of pathological responses, including mitochondrial dysfunction, chronic inflammation, and neuronal damage [19]. Therefore, antioxidants and metal ion chelators have been considered as potential therapeutic strategies for the treatment of AD [20,21,22].

Oxidative stress and neuroinflammation (NI) are interrelated. Chronic and persistent microglial activation, which is stimulated by ROS, leads to an increase in inflammatory mediators (e.g., IL-1β, NO, and TNF-α) and induces NI, thereby creating a neurotoxic environment and accelerating neuronal death [23,24,25,26,27]. Therefore, reducing the levels of proinflammatory mediators and cytokines by inhibiting the hyperactivation of microglia may be a useful approach for the treatment of neuroinflammation-mediated neurodegenerative diseases [28,29].

Increasing evidence shows that the “one molecule, one target” mode of traditional AD treatment strategies has difficulty slowing down AD progression because of the complexity of AD etiology and the diversity of pathogenic factors involved [30,31]. Therefore, developing multi-target-directed ligands (MTDLs) that can simultaneously modulate different targets or mechanisms in the neurodegenerative AD cascade has emerged as a new strategy for the treatment of AD [32,33,34,35].

**Figure 1:**
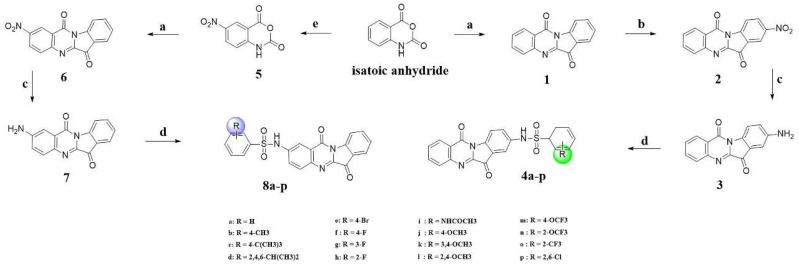
Synthesis route structure of tryptanthrin derivatives: **4a**–**p** and **8a**–**p**. Reagents and conditions: (a) Et_3_N (5 equiv), toluene, reflux, 2–4 h, 70–85%; (b) Concd HNO_3_/H_2_SO_4_, 0 °C, 0.5 h, 95%; (c) SnCl_2_.2H_2_O (2 equiv), ethanol/concd HCl, 80 °C, 2 h, 38%; (d) Pyridine, rt, overnight; (e) Concd H_2_SO_4_, KNO_3_, 0 °C, 1 h, 93%.

Tryptanthrin, found in Radix Isatidis (Ban Lan Gen), is a natural product containing an indoloquinazoline moiety and has the advantages of low toxicity, limited side effects, and a wide range of pharmacological functions, such as neuroprotective activities and antitumor and anti-inflammatory actives [36,37,38]. Currently, a series of new tryptanthrin derivatives and related alkaloid derivatives have been reported to exhibit excellent therapeutic effects in neurodegenerative diseases [39,40]. In our recent study [41], we synthesized a series of novel tryptanthrin derivatives with benzenesulfonamide substituents and evaluated their ameliorating effect on adjuvant-induced arthritis (AIA) rats (Figure 1). All compounds have shown inhibitory effects on NO and inflammatory factors in LPS-induced RAW264.7. The in vivo results indicate that 8j (the best anti-inflammatory activity in vitro) exhibited good anti-inflammatory activity in the AIA model. Moreover, recent studies suggested that anti-inflammatory compounds, which have been proven effective in one particular disease, could also be used in other diseases. Therefore, in this work, we further studied the anti-AD effect of this series of derivatives. We evaluated the anti-ChE properties and neuroprotective effects of this series of derivatives. Furthermore, the target compound **4h** was further evaluated for its potential effects of the inhibition of self-induced Aβ aggregation, inflammatory factors level, ROS release, DPPH, metal chelation properties, and blood–brain barrier (BBB) permeability. In in vivo study, we also investigated whether the target compound could ameliorate the learning and memory impairment of the scopolamine-induced AD mice model.

## 2. Results

### 2.1. Cholinesterase Inhibition Activity

The inhibition ability of tryptanthrin derivatives toward AChE (from electric eel) and BuChE (from equine serum) were determined by Ellman’s assay. Donepezil and tacrine were used as positive controls [42]. As shown in Table 1, most of tryptanthrin derivatives had good inhibitory effects on ChE activity. Among those, compound **4h** showed the strongest inhibitory activity against ChE (AChE, IC_50_ = 0.13 ± 0.04 μM; BuChE, IC_50_ = 6.11 ± 0.45 μM). Therefore, **4h** was selected as the representative compound for further study.

The structure–activity relationship (SAR) analysis showed that the 8-position substitution tryptanthrin derivatives (**4a**–**p**) inhibited cholinesterase significantly more than the 2-position substitution tryptanthrin derivatives (**8a**–**p**). Among **4a**–**p**, the electronegativity of the substituent base on the aromatic ring had a significant effect on the inhibitory activity of the compounds, and the compounds with a strong electron withdrawing group on the benzene ring exhibited significant inhibitory activity for AChE, namely compounds **4f**, **4g**, **4h**, **4m**, **4n**, **4o,** and **4p**. In addition, as the volume of benzene ring substituent increased, the inhibitory activity for AChE decreased markedly. The inhibitory activities of derivatives against AChE gradually increased (IC_50_ value: **4n** = 5.92 ± 0.56 μM, **4o** = 2.45 ± 0.25 μM, **4h** = 0.13 ± 0.04 μM) when -OCF_3_, -CF_3_, -F were introduced into the C2 positions of their benzene rings. The effectiveness of the derivative with -F in the C2 position had a more beneficial effect on AChE inhibitory activity than the C3 or C4 positions (IC_50_ value: **4h** = 0.13 ± 0.04 μM, **4g** = 3.21 ± 0.39 μM, **4f** = 0.56 ± 0.07 μM). This phenomenon was also observed in the derivatives with -OCF_3_ substituents. The derivative with an -OCF_3_ substituent in the C2 position was more effective than that of the derivative with an -OCF_3_ substituent in the C4 position (IC_50_ value: **4n** = 5.92 ± 0.56 μM, **4m** = 7.75 ± 0.21 μM). For BuChE inhibitory activity, the introduction of F also markedly improved the ability of the compounds to inhibit BuChE. However, the introduction of -OCF_3_ (**4n**) at the second position of the benzene ring showed greater inhibitory activity than the introduction of -F (**4h**), likely because the wider active site of BuChE can accommodate larger side-chain groups compared with the long and narrow active site of AChE. Among the **8a**–**p** series, the compounds with electron-donating groups on the benzene ring had significant inhibitory activity for AChE, namely compounds **8a**, **8b**, **8c,** and **8j**. In addition, with the volume of the substituent of benzene ring increased, the inhibitory activity for AChE also decreased markedly. For example, the inhibitory activities of derivatives against AChE gradually increased (IC_50_ value: **8b** = 4.55 ± 0.8 μM, **8c** = 15.84 ± 2.6 μM, **8d** > 50 μM) when -CH_3_, -C(CH_3_)_3_, -2,4,6-CH(CH_3_)_2_ were introduced into the benzene rings. For BuChE inhibitory activity, all the compounds showed negligible inhibitory activity except **8i** and **8j**.

In summary, the SAR analysis provides valuable insights into the structural modifications that can enhance the cholinesterase inhibitory activity of these tryptanthrin derivatives. The results highlight the promising potential of compound **4h** for further investigation.

### 2.2. Reversibility Studies of ***4h*** for AChE/BuChE Inhibition

**Figure 2:**
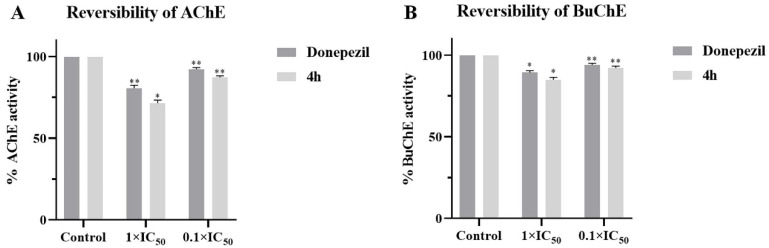
(**A**) AChE and (**B**) BuChE reversibility studies of **4h**. The results are shown as means ± SD (*n* = 3) of at least three independent experiments. * *p* < 0.01, ** *p* < 0.001 compared with control group.

To investigate the reversibility of AChE/BuChE inhibition by the selected compound **4h** (the most active compound), a dilution method was performed [43,44]. Specifically, AChE/BuChE were preincubated with compound **4h** at 10-fold and 100-fold IC_50_ concentrations for 30 min at 37 °C. The reactions solution was then diluted 100-fold to 1 × IC_50_ and 0.1 × IC_50_. The activities of the diluted residual enzymes were measured by Ellman’s modified method. For reversible enzyme inhibitors, the enzymatic activity can be restored to levels of approximately 90% and 50%. Therefore, the obtained results indicate the reversible inhibitor properties of **4h** under experimental conditions (Figure 2 and Table 2).

### 2.3. Kinetic Study of AChE and BuChE Inhibition

**Figure 3:**
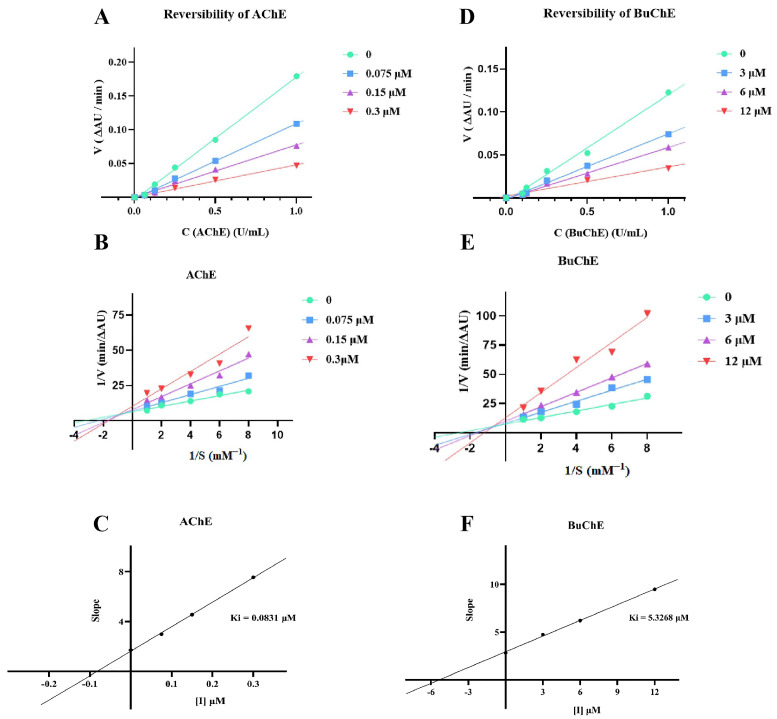
(**A**) Inhibition type of **4h** for AChE. (**B**) AChE of Lineweaver–Burk plot with **4h**. (**C**) hydrolysis and slope replot vs **4h** concentration in fixed AChE. (**D**) Inhibition type of **4h** for BuChE. (**E**) BuChE of Lineweaver–Burk plot with **4h**. (**F**) hydrolysis and slope replot vs **4h** concentration in fixed BuChE. All measurements were measured in triplicate.

To determine the AChE/BuChE enzymatic inhibition type and Ki (inhibition constant), compound **4h** was selected for further kinetic studies [45]. According to the relationship between inhibitors and enzymes, enzyme inhibitors are divided into two categories: irreversible and reversible inhibitors. As shown in Figure 3A,D, whether **4h** was added or not, a straight line through the origin was obtained, and its slope decreased with increasing concentrations of **4h**, which was indicative of reversible inhibition. However, reversible inhibition can be further classified into four types: competitive inhibition, noncompetitive inhibition, anticompetitive inhibition, and mixed inhibition. The result is expressed as a Lineweaver–Burk double-reciprocal plot. As shown in Figure 3B,E, both the slope (decreased Vmax) and intercept (increased Km) of the trend line rose with increasing inhibitor concentration, generally indicative of mixed-type inhibition. Additionally, the dissociation constant (Ki, intercept with the X axis) of the AChE and BuChE inhibitor complex was analyzed by plotting the Lineweaver–Burk secondary plot. As shown in Figure 3C,F, the dissociation constant Ki of **4h** for AChE and BuChE of the compound was determined to be 0.0831 and 5.3268 μM, respectively.

### 2.4. Docking Analysis of ***4h*** with AChE and BuChE

To further investigate the interaction between the ligand (**4h**) and receptor (AChE/BuChE), molecular docking was performed using the CDOCKER molecular docking program to analyze the binding modes. Furthermore, to verify the reliability of the docking results, the cocrystallized ligands were redocked into the active pockets of AChE (PDB: 4EY7) and BuChE (PDB: 5NN0) and compared with their initial docking poses by root mean square deviation (RMSD) calculation [46]. The redocked cocrystallized ligands bound at almost the same position, with RMSD values of 0.226 Å and 0.539 Å by CDOCKER (Figure S1 and Table S1), indicating the docking protocol was reliable.

**Figure 4:**
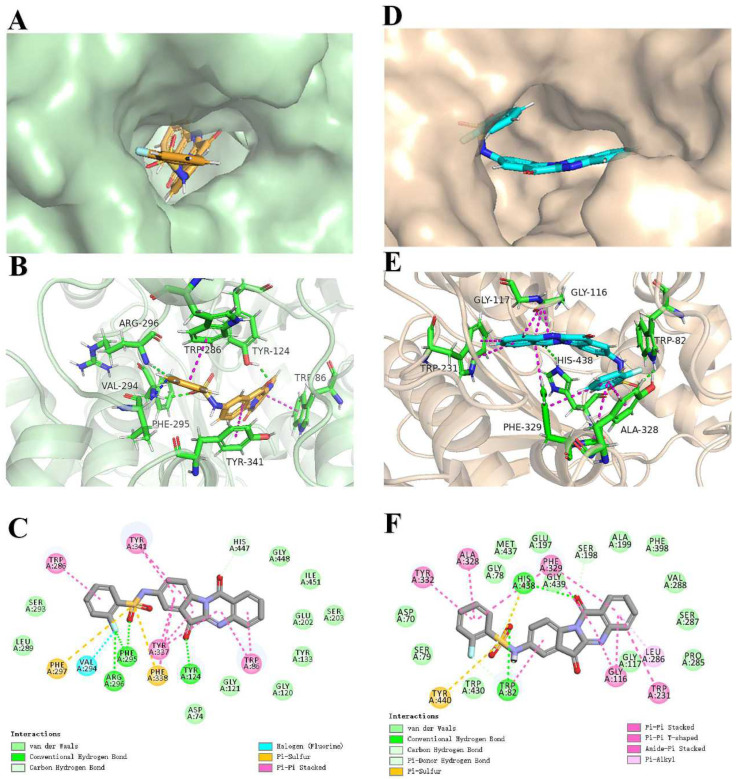
(**A**) Represents surface view of **4h** (yellow) into the active groove site (green) of the AChE (PDB: 4EY7). (**B**) Three-dimensional mode of the interaction of **4h** (yellow) with receptor AChE (green). (**C**) Two-dimensional mode of the interaction of **4h** with receptor AChE. (**D**) Represents surface view of **4h** (blue) into the active groove site (Pink) of the BuChE (PDB: 5NN0). (**E**) Three-dimensional mode of the interaction of **4h** (blue) with receptor BuChE (Pink). (**F**) Two-dimensional mode of the interaction of **4h** with receptor BuChE.

The X-ray cocrystal structure in AChE shows a deep and narrow active site canyon consisting of two separate ligand-binding sites: the peripheral anion-binding site (PAS) and catalytically active site (CAS). The PAS is located near the mouth of the active site canyon, and the aromatic residue Trp 286 is an important anionic site. The CAS contains the AChE catalytic triad Ser 203, Glu 334, His 447 and the important aromatic residue Trp 86 located at the bottom of the canyon [47,48,49]. As shown in Figure 4A–C, **4h** occupied this active site and interacted extensively with both the CAS and PAS. In the **4h**–**AChE** complex, the benzene ring on benzenesulfonyl chloride formed a π–π stacking interaction with the key amino acid residue Trp 286 (the benzene ring of the 2-hydroxyl group, distance = 4.21 Å), while the indole ring generated two π–π stacking interactions with the key amino acid residue Typ 341 (distance = 4.93 Å). The carbonyl oxygen on the indole formed a hydrogen bond with Tyr 124 (distance = 1.89 Å). In addition, the carbonyl oxygen on benzenesulfonyl chloride formed a hydrogen bond with Phe 295 (distance = 2.34 Å), and the fluorine on the benzene ring formed hydrogen bonds with Phe 295 (distance = 2.45 Å) and Arg 296 (distance = 2.19 Å), which enhanced its binding ability to PAS. Within the CAS located in AChE, two π–π stacking interactions were observed between quinoline and the key amino acid residue Trp 86 (distance = 3.75–4.52 Å), along with a hydrocarbon interaction between the carbonyl oxygen on the quinoline ring and His 447 (distance = 2.34 Å).

In the **4h**–**BuChE** complex, the surface view (Figure 4D) of BuChE with **4h** indicates the molecule adopted a U-shaped conformation to fit the available space within the cavity. As illustrated in Figure 4E,F, the key amino acid Trp 82 formed a hydrogen bond with the carbonyl oxygen (distance = 2.31 Å) on benzenesulfonyl chloride and forms a π–π stacking interaction with the benzene ring in the indole ring (distance = 5.53 Å). Additionally, the carbonyl oxygen on the benzothiazole ring formed a hydrogen-bond interaction with the amino acid His 438 (distance = 2.11 Å). Furthermore, hydrophobic interactions were observed between compound **4h** and residues Tyr 332, Ala 328, Phe 329, Gly 116, Tpy 231, and Leu 286 (distance = 3.62–5.65 Å). Thus, the molecular modeling provides valuable insights into the specific binding modes and interactions of compound **4h** with both AChE and BuChE. The extensive interactions observed help explain the potent enzyme inhibitory activities.

### 2.5. Cell Viability and Neuroprotection Study

Prevention of oxidative stress is a major consideration when designing drugs for the treatment of AD [50]. Therefore, compounds **4a**–**p** and **8a**–**p** were selected to evaluate neuroprotective effects against H_2_O_2_-induced PC12 cell injury using 3-(4,5-dimethylthiazol-2-yl)-2,5-diphenyltetrazolium (MTT) assay. Quercetin, a well-known natural antioxidant, was used as a reference compound [50,51]. First, the compounds were analyzed for their potential cytotoxicity with MTT. As shown in Table 3, none of the compounds exhibited evident toxicity at 30 and 50 μM, indicating cytotoxicity did not interfere with the results of the neuroprotection experiments. Next, neuroprotection experiments were conducted based on the cytotoxicity results. As summarized in Table 3, the synthesized compounds showed varying degrees of protection against H_2_O_2_-induced damage in PC12 cells in a concentration-dependent manner compared with the H_2_O_2_-induced control group. Among these compounds, **4g**, **4h**, and **4p** showed significant neuroprotective effects on H_2_O_2_-treated PC12 cells at 30 μM, especially compound **4h**, which had the highest cell survival rate of 71.31 ± 1.9%. This result further supports compound **4h** as the most promising candidate for further investigation.

### 2.6. Effect on Aβ_1-42_ Self-Aggregation

The amyloid hypothesis suggests that Aβ production, oligomerization, and self-aggregation can lead to synaptic dysfunction and age spot formation, as well as accelerating the development of AD [52]. Therefore, we further analyzed the influence of compound **4h** on the self-aggregation of Aβ via thioflavin T (ThT) fluorescence analysis. Donepezil (100 μM) and curcumin (20 μM) were used as positive controls. As shown in Table 4, compound **4h** (100 μM) significantly prevented the self-aggregation of Aβ_1-42_ peptides with an inhibition rate of 63.16% ± 2.33%, superior to the positive drugs donepezil (41.21 ± 1.87%) and curcumin (55.41 ± 2.31%). 

**Figure 5:**
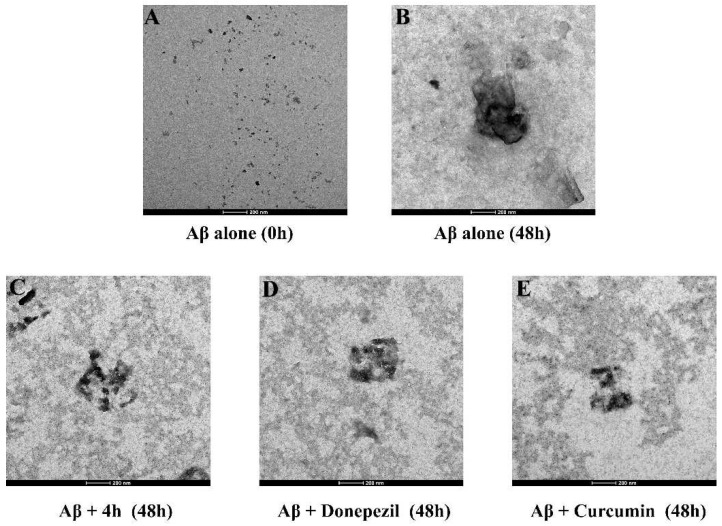
TEM images showing the self-induced aggregation of 5 μM Aβ_1-42_ in the presence or absence of compounds. (**A**) Aβ_1-42_ incubated alone at 0 h. (**B**) Aβ_1-42_ incubated alone at 48 h. (**C**) Aβ_1-42_ and compound **4h** (100 μM) incubated for 48 h. (**D**) Aβ_1-42_ and donepezil (100 μM) incubated for 48 h. (**E**) Aβ_1-42_ and curcumin (20 μM) incubated for 48 h.

The effect of compound **4h** on the morphology of Aβ_1-42_ aggregates was further examined by transmission electron microscopy (TEM). As shown in Figure 5, after 48 h of incubation, most of the Aβ_1-42_ samples were significantly self-aggregated compared with Aβ_1-42_ at 0 h. However, when Aβ_1-42_ was preincubated with compound **4h** for 48 h, the aggregation of Aβ_1-42_ samples was remarkably reduced compared with the positive drug. The TEM results further demonstrate that compound **4h** could inhibit Aβ_1-42_ aggregation and are consistent with the ThT assay results.

### 2.7. Molecular Docking to Aβ_1-42_

**Figure 6:**
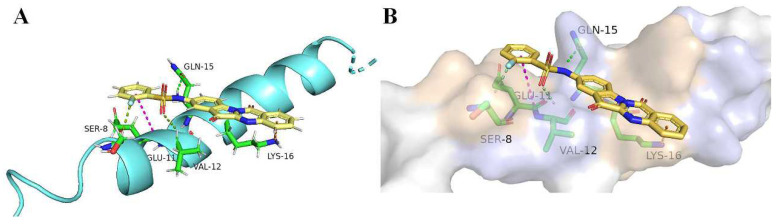
(**A**) Hypothetical conformations of complexes with Aβ_1-42_ (PDB: 1IYT) of **4h**. (**B**) **4h** (yellow) and key residues of Aβ_42_ (green/pink) are shown as sticks. Display receptor surface of **4h** by hydrophobicity. Blue region represents the hydrophobic region while orange region represents the hydrophilic region.

Based on the observed effect of compound **4h** on Aβ self-aggregation, molecular docking experiments were subsequently performed to explore the binding mode between **4h** and Aβ_1-42_ (PDB: 1iyt) [53,54]. As illustrated in Figure 6, the ammonia atom of benzenesulfonamide could interact with important residue via conventional hydrogen bonding, such as Gln 15 (distance = 2.22 Å). In addition, the F and O atoms have carbon–hydrocarbon bonding interactions with amino acids such as Val 12 (distance = 2.85 Å) and Ser 8 (distance = 2.61 Å). Moreover, hydrophobic interactions were displayed between **4h** with Glu 11 (distance = 4.97 Å), which may favor the binding of Aβ_42_ to **4h**. The benzene ring in benzopyrimidine also formed π–cation interaction with amino acid Lys 16 (distance = 3.75 Å). The molecular docking studies indicated that **4h** could effectively bind to Aβ, inhibit the toxic conformation of Aβ_1–42_, and stabilize the α-helical content.

### 2.8. Inhibition of Proinflammatory Cytokines

**Figure 7:**
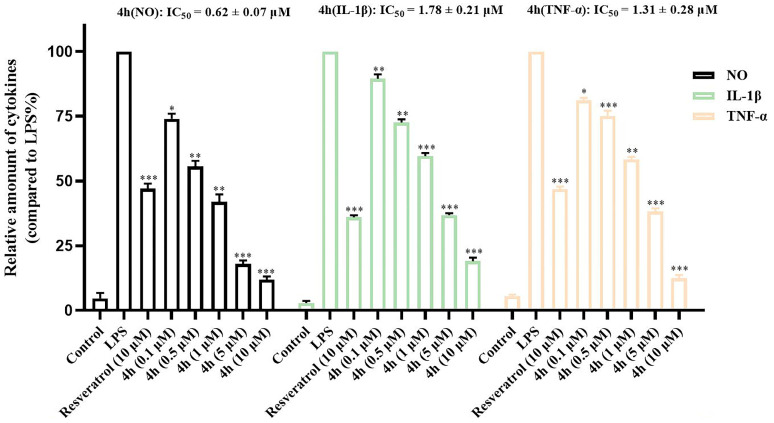
The inhibitory effect of **4h** on the secretion of related proinflammatory factors. BV2 cells were incubated with different concentrations of **4h** (0.1, 0.5, 1, 5, and 10 μM) and the positive drug (Resveratro*l,* 10 μM) for 1 h. The cells were then stimulated by LPS for 24 h. The effects of **4h** on NO, IL-1β, and TNF-α were analyzed by Griess Reagent assay and ELISA method. The results are shown as means ± SD (*n* = 3) of at least three independent experiments. * *p* < 0.001, ** *p* < 0.001, *** *p* < 0.0001 compared with LPS-stimulated cells.

BV2 cells are the main cells involved in neuroinflammation. These cells can be activated by LPS and secrete inflammatory factors such as NO, IL-1β, TNF-α, nitric oxide synthase (iNOS), and cyclooxygenase 2 (COX-2), which mediate the development of various inflammatory diseases [55,56]. Therefore, inhibiting the production of inflammatory mediators in microglia may be an effective treatment approach for neurodegenerative diseases involving neuroinflammation. The inhibitory effect of compound **4h** on the production of inflammatory factors was analyzed by ELISA, using resvertrol as a positive control. As shown in Figure 7, compound **4h** had an evident inhibitory effect on the secretion of inflammatory factors (including NO (IC_50_ = 0.62 ± 0.07 μM), IL-1β (IC_50_ = 1.78 ± 0.21 μM), and TNF-α (IC_50_ = 1.31 ± 0.28 μM)) in BV2 cells compared with the LPS group.

**Figure 8:**
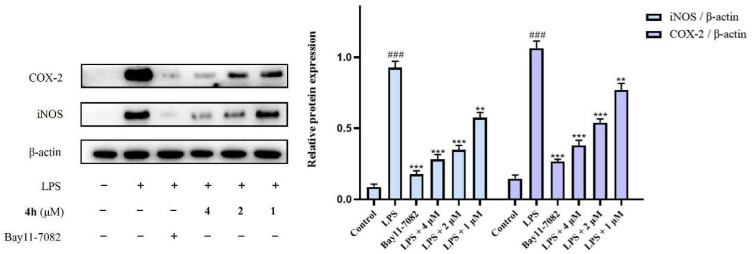
Effects of compound **4h** on the expression of related inflammatory proteins. BV2 cells were incubated with different concentrations of **4h** (1, 2, and 4 μM) and the positive drug Bay 11-7082 (4 μM) for 1 h. The cells were then stimulated by LPS for 24 h. The effects of **4h** on iNOS and COX-2 were analyzed by Western blot. The results are shown as means ± SD (*n* = 3) of at least three independent experiments. ^###^ *p* < 0.0001 compared with control, ** *p* < 0.001, *** *p* < 0.0001 compared with LPS-stimulated cells.

Moreover, the expression of COX-2 and iNOS in LPS-stimulated BV2 cells was analyzed by Western blot. As presented in Figure 8, the relative activities of COX-2 and iNOS were significantly increased in the LPS-induced group versus the control group (LPS). In contrast, compound **4h** significantly decreased the expression of COX-2 and iNOS in a concentration-dependent manner.

### 2.9. Effects of LPS-Induced ROS Production on BV2 Cells

**Figure 9:**
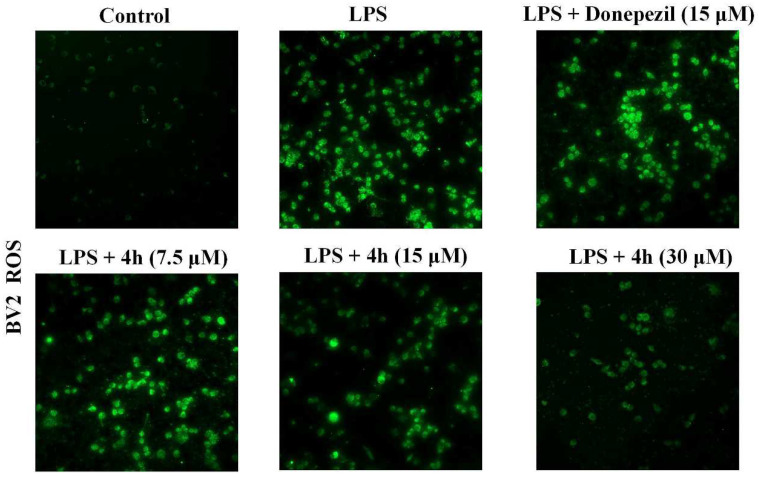
The generation of ROS in BV2 cells was magnified via Confocal laser scanning microscope.

During neuroinflammation, the continuous activation of microglia can not only secrete various proinflammatory factors but also produce excessive intracellular ROS, which can lead to cell death, neuronal damage, and further aggravation of the progression of AD [44,57,58]. Therefore, the inhibitory effect of compound **4h** on LPS-induced ROS production in BV2 cells was investigated using an ROS detection kit. As shown in Figure 9, compound **4h** significantly reduced intracellular ROS levels in BV2 cells in a concentration-dependent manner compared with the LPS-induced group and the donepezil group (15 μM), without affecting BV2 cells morphology. These results indicate that **4h** effectively reduced LPS-stimulated intracellular ROS generation.

### 2.10. Effects on H_2_O_2_-Induced Intracellular Reactive Oxygen Species Production

**Figure 10:**
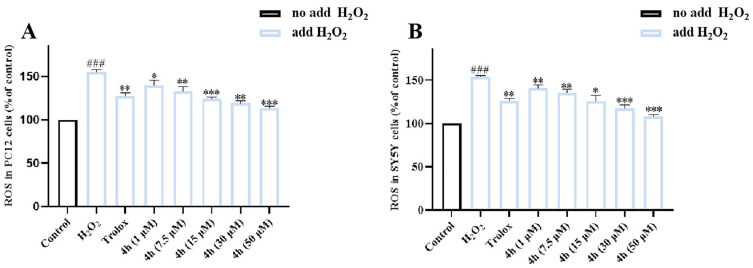
The effect of **4h** on ROS production in H_2_O_2_-induced PC12 cells (**A**) and SY5Y cells (**B**). The results are shown as means ± SD (*n* = 3) of at least three independent experiments. ^###^ *p* < 0.0001 compared with control. * *p* < 0.01, ** *p* < 0.001, *** *p* < 0.0001 compared with H_2_O_2_-induced cells.

Excessive accumulation of intracellular reactive oxygen species (ROS) can lead to oxidative stress and neuroinflammation responses, thereby accelerating the progress of AD. Thus, the effect of the compound **4h** on H_2_O_2_-induced ROS release was evaluated in PC12 or SY5Y cell models using a fluorogenic DCFH-DA probe. In addition, the cytotoxicity assay showed that they had no significant toxicity at 50 μM. Trolox, a water-soluble vitamin E analog, was used as a reference standard [59]. As shown in Figure 10, when the cells are acutely exposed to H_2_O_2_ (without compound), the level of intracellular ROS increases significantly. Notably, compound **4h** significantly reduced the accumulation of ROS in the PC12 (Figure 10A) or SY5Y (Figure 10B) cells in a concentration-dependent manner compared with H_2_O_2_ treatment alone, thereby alleviating the symptoms of AD. These results confirm that compound **4h** effectively inhibits intracellular ROS formation.

### 2.11. DPPH Radical Scavenging Activity of ***4h***

**Figure 11:**
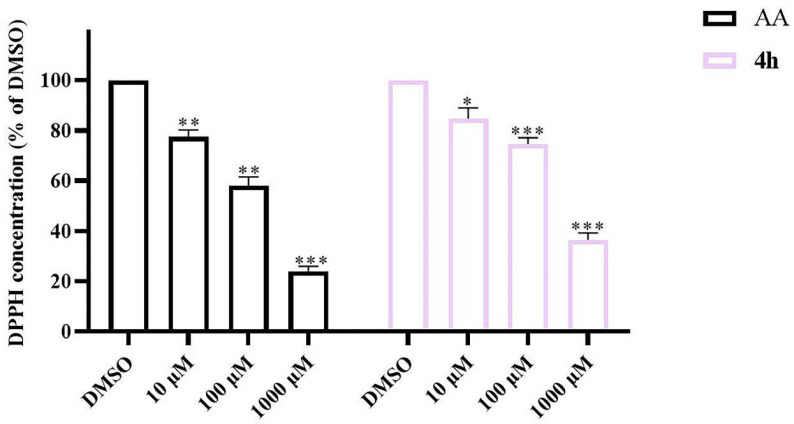
The effect of **4h** on DPPH free radical scavenging. Ascorbic acid (AA) was used as positive controls. The results are shown as means ± SD *(n* = 3) of at least three independent experiments. * *p* < 0.01, ** *p* < 0.001, *** *p* < 0.0001 compared with DMSO.

DPPH is a stable free radical that exists in vitro and is commonly used to screen compounds for their ability to scavenge reactive free radicals. In order to further study the antioxidant properties of the target compound **4h**, DPPH assay was performed with ascorbic acid as the positive control [60]. As shown in Figure 11, from 10 μM to 1000 μM, compound **4h** exhibited moderate free radical scavenging ability compared with ascorbic acid in a dose-dependent manner, with DPPH concentration sharply declining from 84.76% to 36.51%.

### 2.12. Chelating Properties of ***4h***

**Figure 12:**
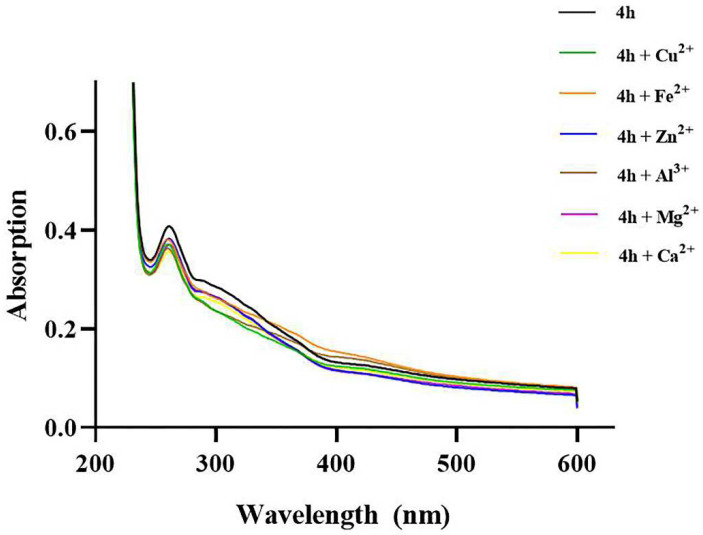
Metal chelation properties of **4h**. UV spectrum of **4h** (20 μM in methanol/water) alone or in the presence of AlCl_3_, CuCl_2_, FeSO_4_, CaCl_2_, MgCl_2_, and ZnCl_2_ (1 equivalent in methanol/water). The ratio of methanol/water is 1/1.

Metal ions are present in Aβ aggregates in AD brains, which can catalyze the production of ROS by participating in the formation of Aβ plaques [61,62]. Therefore, the chelation of antioxidants with biometal ions has become a strategy for the treatment of AD. The metal chelating ability of compound **4h** (methanol/water = 1/1) against biometal ions (Al^3+^, Cu^2+^, Ca^2+^, Fe^2+^, Zn^2+^, and Mg^2+^) was detected by UV–Vis spectroscopy. As shown in Figure 12, upon the addition of CuCl_2_ solution to the compound **4h** solution, the maximum absorption intensity at 261 nm was markedly reduced compared with the untreated blank sample and solutions of other metal ions (AlCl_3_, CaCl_2_, FeSO_4_, ZnCl_2_, and MgCl_2_).

### 2.13. Blood–Brain Barrier (BBB) Permeability

The ability of a drug to cross the BBB to reach its designated site of action is the main indicator used to evaluate its effectiveness in treating various diseases of the central nervous system [63]. P-glycoprotein (P-gp) was highly expressed in BBB microvascular endothelial cells and specifically involved in the efflux transport of drugs at the BBB [64]. MDCK-MDR1 cell lines stably express P-glycoprotein (P-gp) and serve as an in vitro BBB permeation model to distinguish between passive diffusion and P-gp-mediated active efflux [65,66,67]. Therefore, we tested the ability of **4h** to cross the BBB using MDCK-MDR1 cell lines, and bidirectional transport of **4h** was evaluated in apical-to-basal (AP) and basal-to-apical (BL) directions. Diazepam and FD4 were reference compounds for transcellular and paracellular transport, respectively. An efflux ration (ER) value greater than two suggests that a test compound may be a possible substrate for P-gp transport [68]. As shown in Table 5, the apparent permeability of compound **4h** was superior to that of diazepam and the ER value was less than two, indicating suitable BBB permeability.

### 2.14. Metabolic Stability in Liver Microsomes of SD Rats

The adequate metabolic stability of lead compounds in liver microsomal is beneficial for in vivo stability. Therefore, the metabolic stabilities of compound **4h** in rat liver microsomes were evaluated, using donepezil and testosterone as the reference AD positive control agents, respectively [69,70]. According to the results in Table 6, compound **4h** exhibited a longer half-life (T_1/2_ = 108.3 min) in SD rat liver microsomes than donepezil (T_1/2_ = 74.3 min), demonstrating enhanced metabolic stability. Overall, these results reveal that **4h** has suitable in vitro metabolic stabilities to warrant further detailed studies in vivo.

### 2.15. Evaluation of Hepatotoxicity of ***4h*** in AML-12 and HepG2 Cells

**Figure 13:**
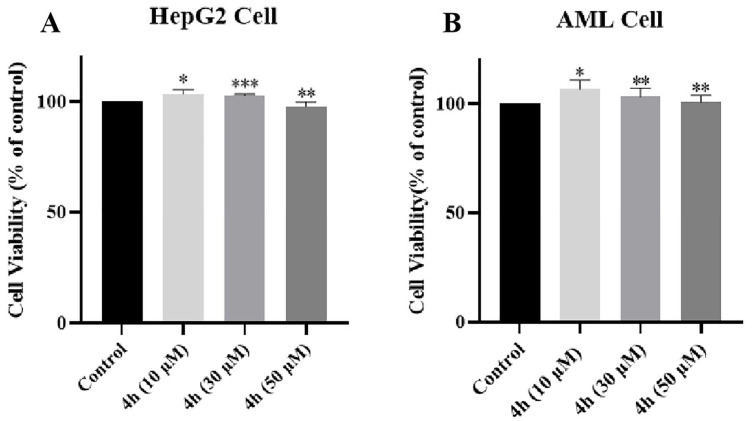
Hepatotoxicity of **4h**. Different concentrations of compounds on cell viability of HepG2 (**A**) and AML-12 cell (**B**) were determined by MTT. The results are expressed as the mean ± SD of three experiments. * *p* < 0.01, ** *p* < 0.001, *** *p* < 0.0001.

The MTT method was used to determine the hepatotoxicity of **4h** in AML-12 and HepG2 cells. Compound **4h** was tested at concentrations of 10, 30, and 50 μM for cytotoxicity to AML-12 and HepG2 cells for 24 h. These results show that **4h** had no toxicity to AML-12 and HepG2 cells at all concentrations (Figure 13A,B).

### 2.16. In Vivo Anti-AD Activity

The improvement of cognitive and memory abilities is important for the development of drugs to treat AD. Therefore, we further evaluated the cognitive effects of compound **4h** in a scopolamine-induced AD mouse model using the Morris water maze test. Scopolamine is known to induce temporary cognitive decline by blocking central cholinergic signaling, mimicking aspects of the cholinergic deficit seen in AD [49,71,72]. The Bliss method was used to calculate the median lethal dose (LD_50_) of **4h**, which was 84.844 (75.72–120.318) mg/kg (95% confidence limit, Table S5). Then, different groups of mice were treated with **4h** (10 and 30 mg/kg, gavage) or donepezil (10 mg/kg, gavage) as a positive control for 7 days.

**Figure 14:**
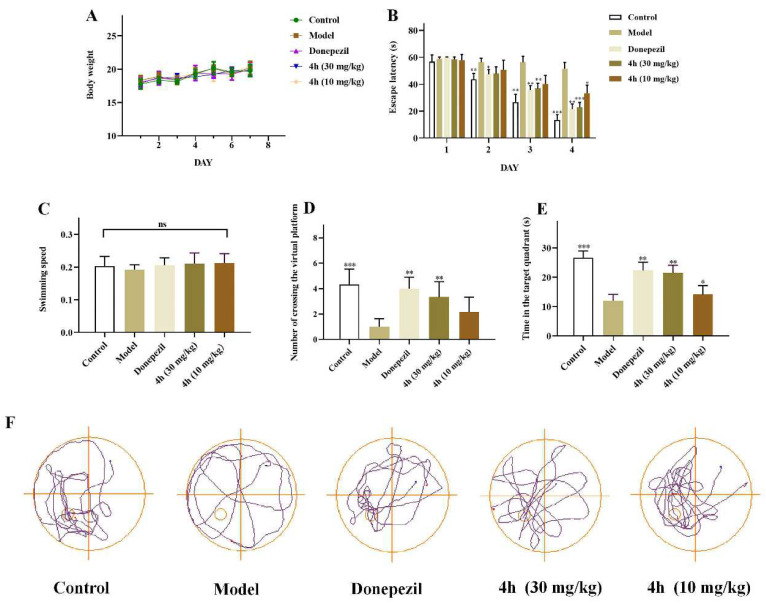
Morris water maze test of **4h** on scopolamine-induced mice. Results are expressed as effect of **4h** plus scopolamine on (**A**) bodyweight. (**B**) Escape latency during the 4-day training period. (**C**) Swimming speed. (**D**) Number of groups crossing the virtual platform. (**E**) Time spent in the target quadrant. (**F**) Representative trajectories of mice in each group during the spatial probe trial period. The mice in the treatment and control groups showed important exploration for hidden platforms in the treatment, whereas the model group displayed random movements. Date are expressed as the mean ± SD (*n* = 7). Statistical significance was analyzed by two-way ANOVA: ^ns^ *p* > 0.5, * *p* < 0.05, ** *p* < 0.01, and *** *p* < 0.001 versus model group.

As shown in Figure 14A, administering different concentrations of **4h** for 7 consecutive days did not cause any abnormal fluctuations in the bodyweight of mice, indicating the doses were well-tolerated. In addition, compared with the model group, no remarkable difference in the escape latency of mice was found in each group, which was about 60 s (Figure 14B). During the following 3 days, the escape latency of the mice in the **4h**-treated group decreased in a dose-dependent manner (see the platform time in Figure 14C). On the fifth day, the platform was removed for the spatial probe trial. The dwell time in the target quadrant and the number of virtual platform crossings were markedly higher in the 30 mg/kg dose group than those in the model and 10 mg/kg dose groups (Figure 14D,E). Their motion tracks also appeared more direct compared with mice in the model group (Figure 14F), indicating better retention of spatial memory. Together, these observations indicate that **4h** has evident therapeutic effects on cognitive impairment and improvement of spatial memory in AD model mice in a dose-dependent fashion.

## 3. Discussion

In this study, we investigated two series of previously designed and synthesized benzenesulfonamide-containing tryptamine derivatives as potential multifunctional anti-AD drugs based on a multi-target-directed ligand strategy. The inhibition of cholinesterase activity to reduce acetylcholine depletion is a major approved strategy for the treatment of AD; so, we first evaluated the effect of the synthesized tryptanthrin derivatives on cholinesterase activity. We found that the derivatives could significantly and nonselectively inhibit both AChE and BuChE, indicating a mixed reversible dual inhibitor profile. They also exhibited an inhibitory effect on amyloid Aβ_1-42_ self-aggregation. Structural activity analyses revealed that halogens on the benzene ring had a significant effect on cholinesterase inhibition, with substituents at the C2 position being the most potent. Compound **4h** (introducing an F group at the 2-position on the benzene ring) displayed the best profile (AChE, IC_50_ = 0.13 ± 0.04 μM; BuChE, IC_50_ = 6.11 ± 0.15 μM), thus being selected as the target compound for further studies.

Extracellular Aβ deposition is a major pathological hallmark of AD. Aβ production, oligomerization, and self-aggregation lead to neurodegeneration [73,74,75]. In addition, AChE induces Aβ aggregation and deposition through the formation of AChE-Aβ complexes, which further contributes to cognitive dysfunction. Therefore, the inhibition of AChE and prevention of Aβ aggregation are considered to be promising therapeutic approaches. Compound **4h** significantly inhibited the self-aggregation of Aβ_1-42_ by 63.16 ± 2.33% compared with the positive drug donepezil, validated by morphological observation of Aβ_1-42_ self-aggregation by TEM.

Neuroinflammation also contributes to the pathogenesis of AD. It is a chronic process that triggers various inflammatory processes in the brain. Activated microglia can secrete large amounts of inflammatory cytokines, such as NO, IL-1β, TNF-α, etc., leading to a neuroinflammatory cascade that causes neuronal death and neurodegenerative diseases, including AD [76,77]. Therefore, the use of anti-inflammatory drugs may reduce the risk of developing AD. We then investigated the effect of the target compound **4h** on the production of inflammatory cytokines in LPS-stimulated BV2 cells to assess its anti-inflammatory activity, showing that **4h** had good anti-inflammatory activity, significantly reduced the production of NO, IL-1β, TNF-α, and downregulated the expression of COX-2 and iNOS in BV2 cells. In addition, the “oxidative stress” hypothesis is another important etiological mechanism in AD which can increase the production of free radicals and reactive oxygen species. It was found that compound **4h** not only significantly reduced intracellular ROS production but also effectively scavenged DPPH free radicals. Based on the abnormal concentration of biogenic metal ions observed in the brains of AD patients, we evaluated the metal chelating ability of **4h** and showed that it can chelate Cu^2+^.

Currently, the development of novel anti-AD drugs faces these two major and serious challenges: the permeability of the blood–brain barrier and potential toxicity issues [78,79,80]. Therefore, we conducted a bidirectional transport study with cytosolic hepatotoxicity assays, and the results show **4h** displayed good BBB permeability and safety profiles to justify in vivo studies. In scopolamine-induced mouse AD model, **4h** significantly improved memory and cognition at safe concentrations for drug administration based on semilethal dose analysis. 

However, the limitation of this study is that cholinesterase inhibition assay shows that **4h** can significantly inhibit electric eel serum AChE and horse serum BuChE, but the effect of **4h** on human cholinesterase is currently unknown and needs to be confirmed by further studies. In addition, we are not sure whether there are other potential targets for the effects of **4h** on AD-related pathological mechanisms, or whether these targets interact with each other, which is another question that deserves further investigation.

Overall, we believe that **4h** has outstanding performance as a multifunctional anti-AD lead compound for further development and that its cascade response to human acetylcholine and AD-related pathological mechanisms needs to be further investigated, and we are confident about its prospect of being developed as a new generation of clinical anti-AD drugs.

## 4. Materials and Methods

### 4.1. Materials

Compounds **4a**–**p** and **8a**–**p** were designed and synthesized by our laboratory in the early period [41]. Commercially available chemical references were used as follows. AChE (from electric eel) and BuChE (from equine serum), acetylthiocholine iodide (ATCI), S-butyrylthiocholine iodide (BTCI), 5,5-dithiobis-2-nitrobenzoic acid (DTNB), DPPH, curcumin, donepezil, scopolamine hydrobromide, 3-[4,5-Dimethyl-2-thiazolyl]-2,5-diphe nyltetrazolium bromide (MTT), and 2′, 7′dichlorofluorescein diacetate (DCFH-DA were obtained from the Sigma Chemical Company (St. Louis, MO, USA). NO, TNF-α, IL-1β ELISA kit, and Aβ_1-42_ solution were bought from the Beyotime Institute of Biotechnology (Haimen, China). All other chemical products and reagents in this study were analytically pure and obtained from approved companies.

### 4.2. Chemistry 

The synthesis of target compounds is outlined in Figure 1. Isatoic anhydride was reacted with isatin in the presence of triethylamine and toluene by one-pot method to yield compound **1** (tryptanthrin). Then, **1** was nitrated in concentrated H_2_SO_4_ in the presence of HNO_3_ to afford compound **2** (8-nitro-substituted tryptanthrin). Compound **3** (8-amino-substituted tryptanthrin) was obtained from compound **2**, which was reduced in ethanol solution under the action of SnCl_2_ and HCl. Finally, in pyridine solvent, compound **3** was reacted with benzenesulfonyl chloride of various substituents by amide substitution reaction and the desired derivatives **4a**–**p** were acquired. However, isatoic anhydride was nitrated in concentrated H_2_SO_4_ in the presence of KNO_3_ to yield compound **5** (5-nitro-isatoic anhydride). Compound **6** (2-nitro-substituted tryptanthrin) was prepared from compound **5** and isatin by one-pot method. Subsequently, compound **7** (2-amino-substituted tryptanthrin) was provided by a reduction of the nitro group through treatment with SnCl_2_ and HCl in EtOH. Finally, it was reacted with various benzenesulfonyl chlorides to acquire the desired derivatives **8a**–**p**.

### 4.3. AChE and BuChE Inhibition Experiments 

The ChE inhibitory activity of the test compounds was determined using the modified Ellman’s method. The compounds were dissolved in DMSO (10 mmol/L) and then diluted with PB (phosphate buffer) solution to attain a range of final concentrations. Donepezil and Tacrine were used as the reference inhibitors. Briefly, 100 μL of different concentrations of the test or standard compounds and 100 μL of AChE/BuChE (0.5 U/mL) were added into 48-well plates and then co-incubated at 37 °C for 30 min. After the end of incubation, 100 μL of substrate (acetylthiocholine iodide or ATCI: 0.35 mM, S-butyrylthiocholine iodide or BTCI: 0.35 mM), 100 μL of PB solution, and 100 μL the color-developing substance (5,5-dithiobis-2-nitrobenzoic acid, DTNB 0.35 mM) were added and then incubated in the dark for 5 min, and absorbance readings at 412 nm were obtained by a multifunctional microplate reader. All concentrations mentioned are final. 

### 4.4. Inhibition Reversibility of AChE/BuChE

Reversibility studies were performed using a modified Ellman’s dilution method to measure the activity of the diluted residual enzyme. The AChE/BuChE (0.5 U/mL), ATCI/BTCI (0.01 M) and DTNB (0.01 M) were diluted with phosphate buffer pH = 7.4. Compound **4h** or donepezil at a concentration of 10 × IC_50_ and 100 × IC_50_ was added to the 96-well plate with 40 μL of enzyme diluent and incubated for 30 min. At the end of incubation, 20 μL of DTNB solution was added to them. Then, the reaction solution was diluted 100 times with the substrate solution to give the final concentrations of inhibitors, 0.1 × IC_50_ and 1 × IC_50_, respectively. Donepezil or buffer was used as the control group. The reaction was further incubated at 37 °C for 15 min. Changes in absorbance were measured at 412 nm by multifunctional microplate reader, and a bar chart was constructed. All the measured results are expressed as the mean ± SEM of the three independent experiments.

### 4.5. Kinetic Studies for AChE/BuChE 

The specific steps are similar to those described in Section 4.3 for the enzyme inhibition assay. In brief, 100 μL of different concentrations of **4h** (0, 0.075, 0.15, and 0.3 μM)/**4h** (0, 3, 6, and 12 μM) and 100 μL of different concentrations of AChE/BuChE (1, 0.5, 0.25, 0.125, and 0.0625 U/mL) were added into 48-well plates and then co-incubated at 37 °C for 30 min. After the end of incubation, 100 μL of ATCI/BTCI (0.35 mM), 100 μL of PB solution, and 100 μL DTNB (0.35 mM) were added, and then changes in absorbance were measured at different times at 412 nm by multifunctional microplate reader, and the catalytic reaction rate of AChE/BuChE was determined. All concentrations are final. With the enzyme reaction rate V as the ordinate and AChE/BuChE concentration as the abscissa, the curve was drawn to determine the reversibility of the compound to AChE/BuChE.

In order to determination of inhibition types and the constant of AChE/BuChE, the concentration of AChE/BuChE (0.5 U/mL) in the reaction system was fixed. Five different substrate concentrations and four different concentrations of test compound **4h** were used to measure the absorbance changes at different times at 412 nm. The reciprocal of the enzymatic reaction rate (1/V) was taken as the ordinate, and the reciprocal of the substrate ATCI/BTCI concentration (1/S) was taken as the abscissa. A Lineweaver–Burk double-reciprocal curve was drawn to determine the inhibition type and Ki (inhibition constant) of compound **4h** on AChE/BuChE. 

Ki = IC_50_/(1 + [S]/Km). Ki is the binding affinity of the inhibitor, IC_50_ is the functional strength of the inhibitor, [S] is the concentration of the substrate, and Km is the concentration of the substrate when the enzyme reaction rate reaches half of the maximum reaction rate, which is the characteristic constant of the enzyme.

### 4.6. Molecular Modeling

The molecular docking was carried out using Discovery Studio 2017 (DS, BIOVIA Software, Inc., San Diego, CA, United States) software in silico procedure to obtain the binding mode of **4h** and the protein. The X-ray crystal structure of the AChE/BuChE/Aβ protein was obtained from the Protein Data Bank (PDB ID: 4EY7, 5NN0, 1IYT). Considering that computer-simulated docking of the compound and different target proteins are the same, **4h** and AChE were taken as examples in this study. First, the ligand was prepared, and the energy was minimized. Subsequently, the original ligand and water molecules were removed in the structure of the AChE protein, and then hydrogen atoms and the CHARMm force fields were added to complete the preparation of the protein. Then, the target compound was docked into the original ligand-binding site of the AChE crystal structure by CDOCKER program. The docking poses of the compound with the highest CDOCKER_ INTERACTION_ENERGY values were selected to analyze the binding mode of the compound to the target protein.

### 4.7. MTT Assay and Neuroprotective Effect against H_2_O_2_-Induced Toxicity

The cytotoxicity and neuroprotective effects of the compounds on PC12 cells were evaluated by MTT assay. PC12 cells were seeded into a 96-well plate at a density of 5.0 × 10^3^ cells per well. After the cells were incubated overnight, the old medium was discarded. The cells were incubated for another 24 h with fresh medium containing compounds at a concentration of 30 or 50 μM. A total of 20 μL of MTT solution (5 mg/mL, Sigma-Aldrich, Shanghai, China) was then added per well, and the cells were incubated for an additional 4 h. Afterwards, the supernatant was removed and 150 μL of DMSO was added to solubilize formazan crystals. The 96-well plate was placed on the shaker for 10–15 min, and then the absorbance at 492 nm was measured by a microplate reader. 

Neuroprotection assay is similar to cytotoxicity. Oxidative cellular damage caused by H_2_O_2_ has become one of the important methods to study oxidative damage in neural cells. PC12 cells were seeded into a 96-well plate at a density of 5.0 × 10^3^ cells per well. After the cells were incubated overnight, the old medium was discarded. The cells were incubated for 3 h with fresh medium containing compounds at a concentration of 30 or 50 μM and then exposed to H_2_O_2_ (250 μM). After incubating overnight, 20 μL of MTT solution (5 mg/mL, Sigma-Aldrich) was added per well, and the cells were incubated for an additional 4 h. Afterwards, the supernatant was removed and 150 μL of DMSO was added. The 96-well plate was placed on the shaker for 10–15 min, and then the absorbance at 492 nm was measured by a microplate reader.

### 4.8. Effect on Aβ_1-42_ Peptide Aggregation

The inhibitory effect of compounds on Aβ_42_ was determined by Thioflavin T (ThT) fluorescence assay. In short, the dilution of Aβ_42_ solution (Beyotime Biotechnology, Hang Zhou, China) and test compounds was carried out with phosphate buffer (50 mM, pH 7.4) to obtain final concentrations of 20 μM of Aβ_1-42_ and 100 μM of test compound, such as curcumin (20 μM). The mixture was incubated in 96-well plates at room temperature for 48 h in the dark. When the incubation was complete, the samples were diluted to 200 µL (final volume) with glycine–NaOH buffer (50 mM, pH 8.0) containing 5 μM ThT. The fluorescence intensity was measured on a Synergy HTX fluorescence microplate reader with excitation and emission wavelengths of λ_ex_ = 450 nm and λ_em_ = 485 nm, respectively. The percentage of inhibition of the self-induced Aβ_1-42_ aggregation for the test compound was calculated by the following formula: (1 − IF_i_/IF_o_) × 100%, in which IF_i_ and IF_o_ are fluorescence intensities in the presence and absence of inhibitors, respectively.

Transmission Electron Microscope (TEM) studies were performed as previously described. Compound **4h** (100 μM) was incubated with Aβ_1-42_ (5 μM), and curcumin (20 μM) and donepezil (100 μM) were chosen as positive drugs. After 2 days, a copper mesh was used to adsorb the samples, then stained with phosphotungstic acid, and the Aβ_1-42_ self-aggregation by TEM was observed (Talos L1200C, Thermo Fisher Scientific, Waltham, MA, USA).

### 4.9. In Vitro Determination of NO, IL-1β, and TNF-α Contents 

The cytotoxicity of compounds on BV2 cells were evaluated by MTT assay and similar to those described in Section 2.5. The NO concentrations in medium were determined using Griess Reagent assay (Beyotime) in accordance with a previously reported method. Nitrite is reduced to nitrogen oxide using Griess Reagent I. Nitrogen oxide then reacts with Griess Reagent II forming a stable product that can be detected by its absorbance at 450 nm. The two-step assay is simple, fast, and can detect nitrite levels as low as 1 nmol/well. BV2 cells were seeded into a 48-well plate at a density of 6.0 × 10^4^ cells per well. After the cells were incubated overnight, the old medium was discarded. Then, the cells were pretreated with various concentrations (10, 5, 1, 0.5, and 0.1 μM) of compounds for 1 h and then co-incubated with LPS (1 μg/mL, 30 μL/well) for 24 h. After 24 h, the cell supernatant (50 μL/well) was placed into a new 96-well plate for experiment. Griess Reagent I and Griess Reagent II (50 μL/well) were mixed at a ratio of 1:1, added to the 96-well plate, and reacted at room temperature for 15 min. The absorbance at 450 nm was measured by a microplate reader. 

The contents of TNF-α and IL-β in the cell supernatant were measured via ELISA (Jyimei, Wuhan, China) following the manufacturer’s instructions.

### 4.10. Western Blot 

BV2 cells were seeded into a 6-well plate at a density of 3.0 × 10^5^ cells per well. After incubation overnight, the old medium was discarded. Then, the cells were pretreated with various concentrations (1, 2, and 4 μM) of **4h** for 1 h and then co-incubated with LPS (1 μg/mL) for 24 h. After incubation was completed, the cells were washed twice with PBS, and 300 μL of cell lysis buffer (RIRA lysis buffer, phosphatase inhibitor, protease inhibitor, and PMSF) was added to each well. The cells were then incubated on ice for 30 min and centrifuged at 12,000 rpm at 4 °C for 30 min to collect the supernatant. The protein content in the supernatant was detected using the BCA protein detection kit (Beyotime, Shanghai, China). Total proteins were separated by 10% SDS-PAGE and then transferred from the gel to a PVDF membrane (Sigma-Aldrich, Shanghai, China). The membranes were blocked in TBST solution containing 5% skimmed milk for 2 h. Next, the membranes were washed three times (TBST) and incubated with primary antibody dilution (Cell Signaling Technologies, Boston, MA, USA) overnight at 4 °C. Then, they were washed another three times (TBST), incubated with the secondary antibody at room temperature for 90 min, and then washed three times with TBST. The blots were visualized by a chemiluminescence imager (Tanon 5200, Shanghai, China). 

### 4.11. ROS Measurement in BV2 Cell

Intracellular ROS levels was detected by the ROS assay kit (Biyuntian Biotechnology, Hang Zhou, China). BV2 cells were divided into 6 groups and seeded in different small dishes, pretreated with tested compounds (**4h** at 30, 15, 7.5 μM and donepezil at 15 μM) for 1 h, and then incubated with LPS for another 24 h. The control group involved culturing the BV2 microglia cells in normal culture media without any treatment. Then, the cells were washed with PBS (pH 7.2) and incubated with DCFH-DA at 37 °C for 20 min. Finally, cells were washed with DMEM and the fluorescence intensity was observed under confocal laser scanning microscope.

### 4.12. Determination of Intracellular ROS Production

A 2’, 7’-dichlorofluorescein diacetate (DCFH-DA, Sigma Aldrich) probe was applied to determine intracellular ROS production. It can penetrate cells and be rapidly oxidized by intracellular ROS to highly fluorescent 2′,7′-dichlorofluorescein (DCF). Intracellular fluorescence intensity is proportional to intracellular ROS levels. PC12 cells or SY5Y cells were seeded into a black 96-well plate at a density of 1.0 × 10^4^ cells per well. After the cells were incubated overnight, the old medium was discarded. PC12 cells or SY5Y cells were cultured in a medium containing test compound **4h** with or without various concentrations (1, 7.5, 15, 30, and 50 μM) for 6 h. Then, the cells were washed with PBS and exposed to H_2_O_2_ (250 μM) for another 24 h. Then, they were washed again with PBS, then DCFH-DA (10 mM) probe was added to each well and incubated at 37 °C for 1 h. After the cells were washed 3 times, the fluorescence intensity was measured in a Synergy HTX fluorescence microplate reader (λ_ex_ = 485 nm, λ_em_ = 528 nm).

### 4.13. Radical Scavenging Activity (DPPH Assay)

DPPH is a commercially available stable organic nitrogen radical with maximum UV absorption at 515 nm. After reduction, the color of the solution fades (absorbance decreases). Therefore, this method was used to evaluate the antioxidant capacity of the compounds. In short, 50 μL of DPPH (140 μM) and 50 μL of different concentrations (10, 100, and 1000 μM) of compound **4h** were added together in a 96-well plate and then incubated in the dark at 37 °C for 2 h. The absorbance was measured at 515 nm through the multifunctional microplate reader. The scavenging rate (SR) of DPPH radicals was calculated by the following formula: SR = (1 − A_c_/A_0_) × 100%, where “A_c_” is the absorbance of DPPH and the compound solution incubating, and “A_0_” is the absorbance of the DPPH radical-ethanol solution alone. The experimental results are expressed as the average of three tests.

### 4.14. Metal Chelating Property

In the wavelength range of 200–600 nm, the metal chelating ability of compound **4h** was analyzed by UV–Vis spectroscopy. Specifically, compound **4h** (dissolved in DMSO) solutions were prepared with a mixture of methanol and deionized water (1/1). Stock solutions of cation salts (10 mM) were prepared with deionized water and then in the presence or absence of ZnCl_2_, CuCl_2_, AlCl_3_, FeSO_4_, MgCl_2_, and CaCl_2_ (20 μM, final concentration), respectively, and incubated with **4h** (20 μM, final concentration) for 1 h at room temperature. The final volume of the reaction mixture was 2 mL. The UV absorption of the test substance was recorded in a 1 cm quartz cell.

### 4.15. Bidirectional Transport Studies

According to the reported method, the BBB penetration of the compound **4h** was evaluated by the well-established MDCKII-MDR1 cell monolayer model. Specifically, MDCKII-MDR1 cells (1 × 10^5^ cell/cm^2^) were seeded on polyester 12-well Transwell inserts (pore size: 0.4 mm, diameter: 12 mm, apical volume: 0.5 mL, basolateral: volume 1.5 mL) and cultured for 5–7 days to reach full differentiation as per TEER readings > 250 Ω·cm. Diazepam and fluorescein isothiocyanate–dextran (FD4) were used as markers for extracellular and paracellular pathways as an internal reference to confirm the integrity of tight junctions during the assay. The compound **4h** solution (75 μM) was added in the AP chamber or in the BL chamber, respectively, for AP-to-BL or BL-to-AP flux studies. After an incubation time of 2 h, samples were removed from the apical and basolateral side of the monolayer and were stored at −80 °C until further analysis. 

The P_app_ was calculated according to the following equation (units of cm/s):

(1) $$P_{app}=\left(\frac{V_{a}}{area\times time}\right)\times \left(\frac{{\left[Drug\right]}_{acceptor}}{{\left[Drug\right]}_{initial}}\right)$$

where “V_a_” is the volume in the acceptor well, “time” is the total transport time, “area” is the surface area of the membrane, “[Drug]_initial_” is the initial drug concentration in the AP or BL chamber, and “[Drug]_acceptor_” is the concentration of the drug in the acceptor chamber.

The ER was calculated using the following equation:

ER = P_app_, BL-AP/P_app_, AP-BL (2)

where “Papp, BL-AP” is the apparent permeability of basal-to-apical transport, and “Papp, AP-BL” is the apparent permeability of apical-to-basal transport.

An efflux ratio greater than 2 indicates that a test compound is likely to be a substrate for P-gp transport.

### 4.16. Metabolic Stability in Liver Microsomes

Compounds **4h**, donepezil, and testosterone were dissolved in CH_3_CN as 10 mM stock solution and incubated with rat liver microsomes (1 mg of protein per mL, final concentration) at a final concentration of 100 μM in a final volume of 0.5 mL of buffer solution (PBS, 100.0 mM; MgCl_2_, 3.0 mM; 1.3 mM β-NADPNa_2_; 3.3 mM glucose 6-phosphate; 0.4 units/mL glucose 6-phosphate dehydrogenase; pH = 7.4). The samples were incubated for various time intervals (0, 5, 10, 30, 60, 90, 120, 150, 180 min) at 37 °C in a water bath. The incubation was terminated at different time points by adding 0.5 mL of ice-cold CH_3_CN. A parallel incubation was performed in the absence of a NADPH-regenerating system with microsomes as the negative control, and the reactions were terminated after the corresponding time incubation. After centrifugation with 12,500 rpm at 4 °C for 10 min, the supernatant was directly analyzed by the HPLC UV systems (Agilent HPLC 1200 instrument). Three independent experiments were performed in triplicate.

### 4.17. Hepatotoxicity Assays

The hepatotoxicity of compound **4h** in normal AML-12 hepatocytes and HepG2 liver cancer cells was examined by MTT method. AML-12 cells and HepG2 cells were seeded into 96-well plates at a density of 5.0 × 10^3^ cells per well, respectively. After the cells were incubated overnight, the old medium was discarded. The cells were incubated for 48 h with fresh medium containing various concentrations of the test compound. After 48 h of incubation, 20 μL of MTT (5 mg/mL) was added to each well and incubate for an additional 4 h. Formazan was dissolved with DMSO (150 μL). The absorbance was measured using a multifunctional microplate reader at a wavelength of 490 nm.

### 4.18. In Vivo Assays

#### 4.18.1. Animals and Treatments

Male C57BL mice (18–23 g; 8 weeks old) were purchased from the Experimental Center of Anhui University of Traditional Chinese Medicine. Mice were reared in a temperature and humidity controlled environment with free access to food and water, with self-adaptive rearing for 4 days. 

#### 4.18.2. Determination of LD_50_ of **4h**

Thirty mice were randomly divided into five groups, and the death of each group was counted after 7 days of intragastric administration (100, 80, 64, 51.2, 40.96 mg/kg). The Bliss method was used to calculate the LD_50_ and 95% confidence limit.

#### 4.18.3. Treatment and Modeling

According to behavioral experiments, 30 mice were randomly divided into 5 groups. The positive control group and the drug group were given donepezil (10 mg/kg) and different concentrations of **4h** (30 mg/kg, 10 mg/kg) by gavage for 7 days, respectively, and the other groups were given 0.5% sodium carboxymethyl cellulose (CMC-Na) solution by gavage for 7 days. On day 7, scopolamine hydrobromide was injected intraperitoneally into the of the mouse to establish an AD model. The control group were only injected normal saline. After 30 min, the bodyweight changes in mice during the administration period were recorded. Scopolamine hydrobromide, donepezil, and CMC-Na were purchased from Aladdin Reagent Company.

#### 4.18.4. Morris Water Maze Test

The MWM is a white circular pool (150 cm in diameter, 60 cm in height; no features on the inner surface) with a water height of 46 cm and a white platform (10 cm in diameter) submerged 1 cm below the water surface. White titanium dioxide (0.25 g/L) was added to make the water turbid to make the platform invisible, and the platform was fixed in the third quadrant of the circular pool (four quadrants in total). Training was performed by allowing mice to stay for 15 s after reaching the platform. If the mice successfully found the platform within 60 s, they were allowed to stay on the platform for 15 s; otherwise, they were brought to the platform and stayed for 15 s. The training was performed four times a day for a total of 4 days of training. Except for the control group, animals were intraperitoneally injected with scopolamine 90 min before the test to induce cognitive deficits mimicking. On day 5, a probe trial was conducted with the platform removed. Mice were placed in the quadrant opposite the target for 60 s, and their swim path was recorded to evaluate memory retention. Performance measures included latency to reach the platform location and time spent in each quadrant.

### 4.19. Statistical Analysis

Data are expressed as the mean ± SEM of at least three independent experiments, and GraphPad Prism 8.01 software was used for statistical analysis.

## 5. Conclusions

In this study, we evaluated two series of tryptophan derivatives following the multi-target-directed ligands strategy as potential anti-AD multifunctional agents. Compound **4h** stood out for further investigation based on its biological profile. **4h** remarkably inhibited the activity of cholinesterase and had a prominent effect on Aβ self-aggregation. Enzyme kinetic analysis showed that **4h** stood out as a mixed-type reversible dual inhibitor of AChE and BuChE. Molecular docking studies provided insights into the interactions of **4h** with AChE, BuChE, and Aβ. In addition, **4h** exhibited good inhibitory activity against the production of inflammatory factors and significantly suppressed the expression levels of iNOS and COX-2. Further study showed that **4h** not only significantly reduced the LPS-mediated accumulation of ROS in BV2 cells but also greatly enhanced the H_2_O_2_-induced survival of PC12 cells and SY5Y cells. Furthermore, **4h** concentration-dependently scavenged DPPH radicals and selectively chelated biometal ion Cu^2+^. Bidirectional transport studies showed that compound **4h** had good BBB permeability. Finally, in vivo studies demonstrated that **4h** could effectively ameliorate cognitive impairment in scopolamine treatment. These findings collectively highlight **4h**’s multitarget profile and support its potential as an innovative disease-modifying agent for Alzheimer’s treatment. Further studies are merited to optimize **4h**’s pharmacodynamics and pharmacokinetics, as well as validate its mechanisms of action in human systems. With continued advancement, **4h** may be developed as a new-generation clinical anti-Alzheimer’s drug.

## Figures and Tables

**Table 1:** Inhibitory activity of compounds **4a**–**p**, **8a**–**p** on ChEs.

Compd	AChE Inhibition, (IC_50_, μM) ^a^	BuChE Inhibition, (IC_50_, μM) ^b^	Selective Index ^c^
**4a**	0.97 ± 0.16	9.98 ± 0.64	10.3
**4b**	5.75 ± 0.27	8.61 ± 1.2	1.5
**4c**	2.56 ± 0.32	8.13 ± 1.21	3.2
**4d**	>50	>50	N/A
**4e**	33.01 ± 1.91	>50	N/A
**4f**	0.56 ± 0.07	11.87 ± 1.86	21.2
**4g**	3.21 ± 0.39	21.58 ± 1.79	6.7
**4h**	0.13 ± 0.04	6.11 ± 0.15	47
**4i**	8.8 ± 1.23	>50	N/A
**4j**	>50	>50	N/A
**4k**	25.41 ± 1.53	>50	N/A
**4l**	18.56 ± 1.77	>50	N/A
**4m**	7.75 ± 0.21	10.19 ± 1.24	1.7
**4n**	5.92 ± 0.56	4.93 ± 0.35	0.6
**4o**	2.45 ± 0.25	8.61 ± 1.2	3.5
**4p**	0.62 ± 0.09	>50	N/A
**8a**	14.02 ± 1.67	>50	N/A
**8b**	4.55 ± 0.8	>50	N/A
**8c**	15.84 ± 2.6	>50	N/A
**8d**	>50	>50	N/A
**8e**	>50	>50	N/A
**8f**	43 ± 1.12	>50	N/A
**8g**	>50	>50	N/A
**8h**	>50	>50	N/A
**8i**	>50	25.87 ± 2.38	N/A
**8j**	>50	10.19 ± 2.41	N/A
**8k**	>50	>50	N/A
**8l**	>50	>50	N/A
**8m**	>50	>50	N/A
**8n**	42.02 ± 1.7	>50	N/A
**8o**	8.86 ± 0.76	>50	N/A
**8p**	>50	>50	N/A
Donepezil	0.021 ± 0.005	7.29 ± 0.6	347.1
Tacrine	0.270 ± 0.064	0.035 ± 0.05	0.12

^a^ AChE from electric eel. IC_50_ values are calculated from the average of three experiments (mean ± SD). ^b^ BuChE from equine serum. IC50 values are calculated from the average of three experiments (mean ± SD). ^c^ SI (Selectivity Index): the AChE selectivity index is defined as IC50 (BChE)/IC50 (AChE).

**Table 2:** The reversibility studies of AChE ^a^ and the reversibility studies of BuChE ^a^.

	AChE		BuChE	
	Donepezil	4h	Donepezil	4h
Control	100 ± 0.01	100 ± 0.01	100 ± 0.01	100 ± 0.01
1 × IC_50_	83.1 ± 1.27	72.3 ± 1.03	89.4 ± 1.49	84.9 ± 1.2
0.1 × IC_50_	92.3 ± 0.74	87.2 ± 0.92	94.1 ± 0.61	92.2 ± 0.52

^a^ All measurements were measured in triplicate, expressed as mean ± SD.

**Table 3:** Cell viability and neuroprotective effect H_2_O_2_-induced PC12 cell death.

Compd	H_2_O_2_-Induced PC12 Cell Viability (% of Control) ^a^			Cell Viability (% of Control) ^a^	
	H_2_O_2_	10 μM	30 μM	30 μM	50 μM
**4a**	32.1 ± 0.9	43.29 ± 3.1	48.5 ± 1.5	99.5 ± 1.7	98.3 ± 3.0
**4b**	32.41 ± 1.1	47.36 ± 1.7	59.09 ± 2.5	99.7 ± 0.9	99.5 ± 1.5
**4c**	30.3 ± 1.6	45.59 ± 0.7	56.4 ± 1.2	99.5 ± 3.1	98.2 ± 1.7
**4d**	32.12 ± 1.2	42.53 ± 2.7	50.1 ± 0.8	99.4 ± 2.4	97.3 ± 3.6
**4e**	38.17 ± 1.6	42.8 ± 1.5	57.51 ± 0.8	99.6 ± 2.1	99.5 ± 2.3
**4f**	34.69 ± 1.4	45.23 ± 2.2	55.26 ± 0.5	99.4 ± 1.6	98.7 ± 1.1
**4g**	36.26 ± 1.9	59.88 ± 0.9	68.83 ± 2.7	100 ± 1.3	99.7 ± 0.8
**4h**	30.1 ± 2.1	61.66 ± 1.3	71.31 ± 1.9	99.6 ± 0.9	99.2 ± 1.5
**4i**	33.92 ± 3.1	34.43 ± 5.5	40.93 ± 2.9	99.6 ± 1.7	98.3 ± 3.0
**4j**	35.4 ± 2.3	38.24 ± 3.1	41.69 ± 1.7	99.7 ± 1.6	99.1 ± 1.2
**4k**	34.22 ± 1.7	44.35 ± 1.5	46.23 ± 0.6	98.8 ± 1.9	98.2 ± 3.4
**4l**	30.9 ± 1.2	36.35 ± 1.6	39.84 ± 2.8	100.2 ± 3.1	99.8 ± 2.7
**4m**	31.6 ± 2.3	37.89 ± 4.3	44.98 ± 2.5	99.6 ± 0.8	99.3 ± 2.1
**4n**	31.5 ± 0.4	46.53 ± 1.2	52.84 ± 1.8	99.9 ± 0.7	99.7 ± 1.8
**4o**	29.31 ± 1.5	42.32 ± 0.7	50.5 ± 0.5	99.6 ± 2.7	98.2 ± 1.7
**4p**	33.9 ± 1.7	48.49 ± 4.5	65.6 ± 2.2	99.6 ± 2.7	99.3 ± 2.9
**8a**	33.77 ± 1.8	39.65 ± 3.1	50.98 ± 1.8	100.1 ± 1.5	99.8 ± 1.7
**8b**	32.9 ± 2.1	42.01 ± 1.7	51.37 ± 2.1	101.2 ± 3.1	99.8 ± 2.4
**8c**	32.16 ± 2.2	39.10 ± 4.5	49.64 ± 4.1	98.4 ± 1.9	96.7 ± 1.9
**8d**	35.33 ± 1.9	39.65 ± 5.2	53.26 ± 1.4	99.8 ± 0.7	98.7 ± 2.6
**8e**	32.6 ± 0.6	41.78 ± 1.2	55.86 ± 0.9	99.8 ± 0.7	99.7 ± 3.1
**8f**	30.72 ± 0.9	34.24 ± 3.5	42.25 ± 1.7	99.4 ± 1.7	98.3 ± 3.0
**8g**	34.41 ± 0.8	44.37 ± 0.5	52.28 ± 2.3	99.6 ± 1.2	99.5 ± 2.7
**8h**	32.91 ± 1.4	36.03 ± 3.0	46.73 ± 3.5	99.4 ± 1.4	98.3 ± 2.7
**8i**	29.9 ± 2.3	48.46 ± 1.6	59.63 ± 2.5	99.8 ± 1.2	99.4 ± 1.3
**8j**	31.7 ± 0.3	38.02 ± 3.1	47.36 ± 6.1	99.9 ± 1.5	98.9 ± 2.1
**8k**	35.44 ± 0.8	44.37 ± 0.5	52.28 ± 2.3	99.6 ± 1.2	99.5 ± 2.7
**8l**	32.83 ± 1.7	34.61 ± 3.7	38.1 ± 2.7	99.4 ± 1.1	98.7 ± 1.3
**8m**	30.13 ± 1.5	44.67 ± 2.3	53.92 ± 3.9	99.5 ± 3.1	98.2 ± 1.7
**8n**	32.21 ± 2.1	38.94 ± 1.5	49.96 ± 0.8	99.2 ± 1.9	98.3 ± 2.7
**8o**	35.1 ± 3.1	42.56 ± 2.1	54.6 ± 3.8	99.7 ± 2.4	99.4 ± 1.1
**8p**	29.33 ± 1.5	42.32 ± 0.7	50.5 ± 0.5	99.6 ± 2.7	98.2 ± 1.7
Quercetin	30.4 ± 0.7	55.21 ± 1.6	63.27 ± 2.1	99.6 ± 1.1	99.5 ± 1.3

^a^ Cell viability was determined using MTT assay protocol. Data are expressed as the mean ± SEM of the three independent experiments.

**Table 4:** The effect of **4h** on Aβ aggregation.

Compd	Inhibition of Self-Induced Aβ Aggregation (%) ^a^
**4h**	63.16 ± 2.33
Curcumin	55.41 ± 2.31
Donepezil	41.21 ± 1.87

^a^ In the presence of 5 μM Aβ and 100 μM inhibitor, the inhibition of Aβ self-aggregation was determined by the ThT fluorescence analysis. Data are expressed as the mean ± SEM of three independent experiments, each performed in duplicate.

**Table 5:** Bidirectional transport of **4h** across MDCKII-MDR1 cells.

Compd	P_app_ AP (×10^−5^cm/s) ^a^	P_app_ BL (×10^−5^cm/s) ^a^	ER ^b^ P_app_ BL/P_app_ AP
**4h**	1.81 ± 0.27	1.74 ± 0.22	0.96
Diazepam	1.36 ± 0.17	1.19 ± 0.15	0.89
FD4	0.47 ± 0.15	0.32 ± 0.14	0.65

^a^ Data are means ± SD of three determination. ^b^ Efflux ratio (ER) was calculated using the following equation: ER PappBL/PappAP.

**Table 6:** Metabolic Stability of Compound in Liver Microsomes of SD Rats.

Compd.	K (min^−1^)	T_1/2_ (min) ^a^
Testosterone ^b^	0.2593 ± 0.04409	2.4 ± 0.5
Donepezil ^b^	0.00834 ± 0.00044	74.3 ± 5.3
**4h**	0.00574 ± 0.00037	108.3 ± 4.9

^a^ Results are expressed as the mean ± SD of at least three independent experiments performed in triplicate. ^b^ The positive control (testosterone) and the compound donepezil exhibited metabolic stability that was consistent with the literature and internal validation data.

## Data Availability

Data are contained within the article.

## Supplementary Materials

The Supplementary Materials information can be downloaded at https://www.mdpi.com/article/10.3390/ph16101468/s1. Heavy Atom RMSD to the conformation of co-ligand (CDOCKER); The effect of compound 4h on BV2, PC12 and SY5Y cells viability in vitro cytotoxicity study; The effect of compound **4h** on ROS production in H_2_O_2_-induced PC12 and SY5Y; The effect of compound **4h** on DPPH free radical scavenging; LD_50_ determination results of compound **4h** on mice after intragastric administration for 7 days.
