# Supplementary material for: Discovery of Novel Tryptanthrin Derivatives with Benzenesulfonamide Substituents as Multi-Target-Directed Ligands for the Treatment of Alzheimer’s Disease
Source: Pharmaceuticals (Basel). 2023 Oct 16;16(10):1468. doi: 10.3390/ph16101468 (PMC10610214; doi:10.3390/ph16101468)
Supplement: Supplementary file 1 [file pharmaceuticals-16-01468-s001.zip › pharmaceuticals-2625456-supplementary.pdf]

SUPPORTING INFORMATION

Discovery of novel tryptanthrin derivatives with benzenesulfonamide substituents as multi-target-directed ligands for the treatment of Alzheimer's disease

Contents

Heavy Atom RMSD to the conformation of co-ligand (CDOCKER)..**2-3**

The effect of compound **4h** on BV2, PC12 and SY5Y cells viability *in vitro* cytotoxicity study...................**4**

The effect of compound **4h** on ROS production in H_2O_2-induced PC12 and SY5Y..**5**

The effect of compound **4h** on DPPH free radical scavenging...**6**

LD_{50} determination results of compound **4h** on mice after intragastric administration for 7 days...................**7**

Figure and Table of contents:

Figure S1 and Table S1. Heavy Atom RMSD to the conformation of co-ligand (CDOCKER).

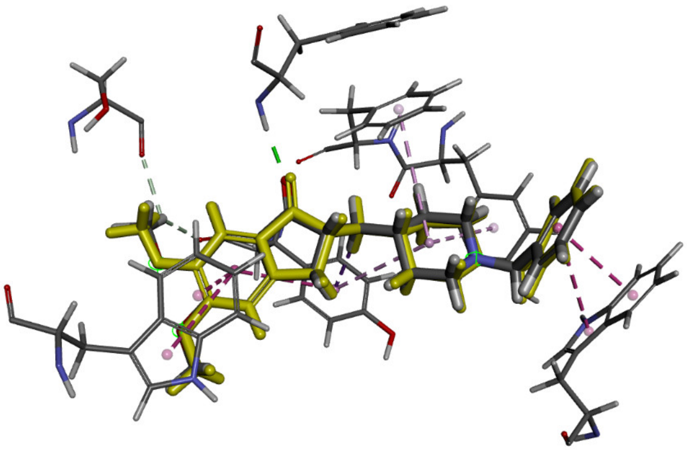

AChE:

Comformation	[a] RMSD (Å)	Comformation	[a] RMSD (Å)
**1**	**0. 226**	7	1.827
2	1.287	8	2.045
3	1.493	9	2.416
4	1.545	10	3.215
5	1.594	co-ligand	0
6	1.775		

[a] RMSD: root mean square deviation.

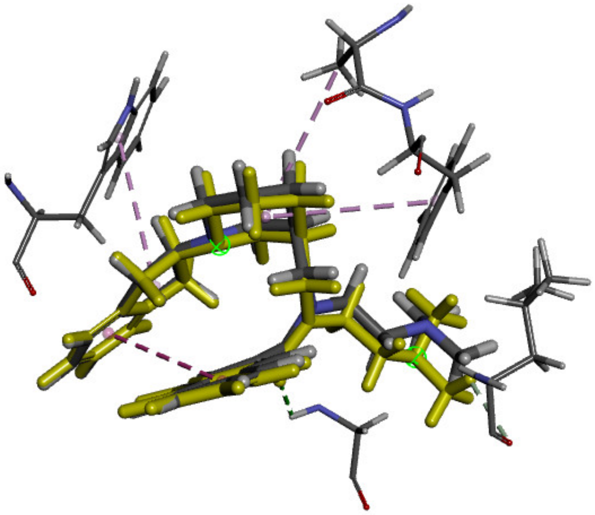

BuChE:

Comformation	[a] RMSD (Å)	Comformation	[a] RMSD (Å)
**1**	**0. 539**	7	4.299
2	0.733	8	5.670
3	1.693	9	5.781
4	2.947	10	7.182
5	3.489	co-ligand	0
6	4.013		

[a] RMSD: root mean square deviation.

Table S2. The effect of compound **4h** on BV2, PC12 and SY5Y cells viability *in vitro* cytotoxicity study.

Cell viability (% of control) [a]	Control	30 μM	50 μM
BV2	100	101.23 ± 1.82	97.44 ± 1.36
PC12	100	99.62 ± 0.94	99.21 ± 1.57
SY5Y	100	97.84 ± 1.24	91.33 ± 1.48

[a] Cell viability was determined using MTT assay protocol. Data are expressed as the mean ± SEM of the three independent experiments.

Table S3. The effect of compound **4h** on ROS production in H_2O_2-induced PC12 and SY5Y.

	ROS in PC12 cells[a] (%)	ROS in SY5Y cells[a] (%)
Control	100	100
Trolox (100 μM)	127.33 ± 3.51	126.06 ± 2.68
**4h** (1 μM)	139.83 ± 5.48	140.33 ± 4.04
**4h** (7.5 μM)	132.96 ± 4.08	135.32 ± 2.32
**4h** (15 μM)	124.17 ± 1.83	125.38 ± 6.18
**4h** (30 μM)	119.83 ± 3.25	117.35 ± 3.6
**4h** (50 μM)	114.13 ± 2.07	108.30 ± 1.65
H_2O_2 group	155.2 ± 3.45	151.76 ± 1.85

[a] The percentage of intracellular ROS compared with the control (untreated control cells). The results were calculated from the average of three experiments (mean ± SD).

Table S4. The effect of compound **4h** on DPPH free radical scavenging.

compd	Concentration of DPPH [a] (%, 10 μM)	Concentration of DPPH [a] (%, 100 μM)	Concentration of DPPH [a] (%, 1000 μM)
**4h**	84.76 ± 4.21	74.6 ± 2.51	36.51 ± 2.78
AA	77.43 ± 2.76	58.10 ± 3.42	24.2 ± 1.85

[a] The concentration of DPPH in the experimental group compared with the no drug or compound group. The results were calculated from the average of three experiments (mean ± SD).

Table S5. LD_{50} determination results of compound **4h** on mice after intragastric administration for 7 days.

Group	Dose (mg/kg)	Number of deaths	Mortality rate (%)
1	100	4	66.7
2	80	3	50
3	64	1	16.7
4	51.2	0	0
5	40.96	0	0
